# Genetic variants of the GLP-1R gene affect the susceptibility and glucose metabolism of gestational diabetes mellitus: a two-center nested case‒control study

**DOI:** 10.1186/s13098-022-00963-1

**Published:** 2022-12-17

**Authors:** Ping Luo, Ying Fan, Yusha Xiong, Hua Feng, Zhiping Yang, Chunlin Zhang, Bing Mei

**Affiliations:** 1 Department of Laboratory Medicine, Jingzhou Hospital Affiliated to Yangtze University, Jingzhou, 434020 China; 2 Gongan County Maternal and Child Health Care Hospital, Jingzhou, 434300 China

**Keywords:** Gestational diabetes mellitus, Glucagon-like peptide-1 receptor, Incretin effect, Type 2 diabetes, Single nucleotide polymorphism

## Abstract

**Background:**

Gestational diabetes mellitus (GDM) is the most common complication during pregnancy, occurring under the combined action of environmental and genetic factors. Genetic variants of glucagon-like peptide-1 receptor (GLP-1R) have been reported to affect insulin secretion and susceptibility to type 2 diabetes. This study aimed to explore the role of GLP-1R polymorphisms in GDM and glucose metabolism.

**Methods:**

A two-center nested case‒control study was designed, including 200 pregnant women with GDM and 200 pregnant women without GDM genotyped for five tag SNPs of GLP-1R using Sanger sequencing. Logistic regression was used to evaluate the relationship between GLP-1R polymorphisms and GDM risk. Glucose and insulin concentrations were measured based upon the 75 g oral glucose tolerance test (OGTT). Beta cell function of different genotypes was estimated with the 60 min insulinogenic index (IGI_60_) and OGTT-derived disposition index (DI).

**Results:**

Mutant genotype AG + GG of tag SNP rs6458093 nominally increased GDM risk (*p* = 0.049), especially among subjects younger than 35 years (*p* = 0.024) and with BMI no less than 24 (*p* = 0.041), after adjusting for confounders. Meanwhile, compared with subjects with wild genotype AA, subjects with genotype AG + GG of rs6458093 also showed nominally significantly lower IGI_60_ (*p* = 0.032) and DI (*p* = 0.029), as well as significantly higher 75 g OGTT-based 1 h glucose load plasma glucose levels (*p* = 0.045). Moreover, the mutant heterozygous genotype GA of tag SNP rs3765467 nominally decreased GDM risk among subjects older than 35 years (*p* = 0.037) but showed no association with insulin secretion and glucose homeostasis.

**Conclusions:**

Tag SNP rs6458093 of GLP-1R was nominally associated with increased GDM risk and affected beta cell function and postprandial glucose metabolism, while tag SNP rs3765467 of GLP-1R was nominally associated with decreased GDM risk, providing evidence for molecular markers and etiological study of GDM.

## Introduction

Gestational diabetes mellitus (GDM) is one of the most common complications during pregnancy, affecting 14% of pregnant women worldwide [1]. The prevalence of GDM among pregnant Chinese women increased remarkably from 4% in 2010 to 21% in 2020 [2]. Macrosomia and neonatal hypoglycemia are the most common complications of GDM [3]. In addition, women with a history of GDM are more likely to develop type 2 diabetes mellitus (T2DM) [4] and cardiovascular disease [5], while their offspring are more likely to become obese [6] and develop T2DM [7] in the future.

Insulin resistance and pancreatic beta cell (β cell) dysfunction are considered to be the main pathogenesis of GDM. To meet the energy needs of the growing fetus by limiting the mother's consumption of glucose, insulin sensitivity decreases due to hormones, obesity, the placenta, cytokines, adipokines, decreased physical activity and so on [8]. Pregnant women with normal glucose tolerance (NGT) can compensate for increasing insulin resistance by increasing the secretion of insulin, while women with GDM have insufficient insulin secretion [9]. In other words, excellent β cell function secreting insulin plays a crucial role in glucose homeostasis during pregnancy.

It is well known that food can stimulate intestinal mucosa L cells to release incretin hormones, which stimulate insulin secretion in a glucose-dependent manner, a phenomenon known as the “incretin” effect [10, 11]. Insulin secretion stimulated by a meal is partly due to direct actions of blood glucose on β cells, while up to two-thirds of postprandial insulin is due to the incretin effect [11, 12]. Glucagon-like peptide-1 (GLP-1) is one of the most important incretin hormones [13], acting through glucagon-like peptide-1 receptor (GLP-1R) signaling. It is reasonable to assume that dysfunction of GLP-1R may affect the function of β cells secreting insulin by inhibiting the interaction between GLP-1 and GLP-1R. Although the development of GDM is closely associated with dysfunction of β cells, the relationship between GDM susceptibility and GLP-1R genetic variation that directly influences the quality or quantity of GLP-1R and β cell function remains unknown.

A previous study found that two SNPs (rs6923761 and rs3765467) in GLP-1R could alter insulin secretion in response to exogenous GLP-1 [14]. In recent years, rs3765467 and rs10305492 of the GLP-1R gene were reported to affect β cell function and promote apoptosis of β cells [15]. Meanwhile, rs3765467 was identified as a susceptibility locus for T2DM in the Japanese population in a meta-analysis of 4 genome-wide association studies (GWAS) [16], and rs10305492 was reported to be linked to lower fasting plasma glucose (FPG) and T2DM risk [17, 18]. Moreover, rs1042044 and rs367543060 of the GLP-1R gene were reported to be associated with susceptibility to T2DM [19, 20]. It is well known that GDM and T2DM share the same pathogenesis (insulin resistance and pancreatic β cell dysfunction) [21] and share a common genetic background [22]. The percentage of GDM women diagnosed with T2DM was 12% higher for each additional year after pregnancy [23]. The estimated risks for T2DM were 19.72% at 10 years, 29.36% at 20 years, 39.00% at 30 years, 48.64% at 40 years, and 58.27% at 50 years [24]. Therefore, GDM is believed to be the early stage of T2DM [25]. The above studies provided a rationale for the hypothesis that GLP-1R polymorphisms might be involved in the pathogenesis of GDM. Herein, we validated the correlation of GLP-1R gene polymorphisms with GDM risk and evaluated the effects of GLP-1R polymorphisms on β cell function and glucose homeostasis to provide preliminary evidence for the genetic and etiological study of GDM.

## Materials and methods

### Study design and participants

**Fig. 1:**
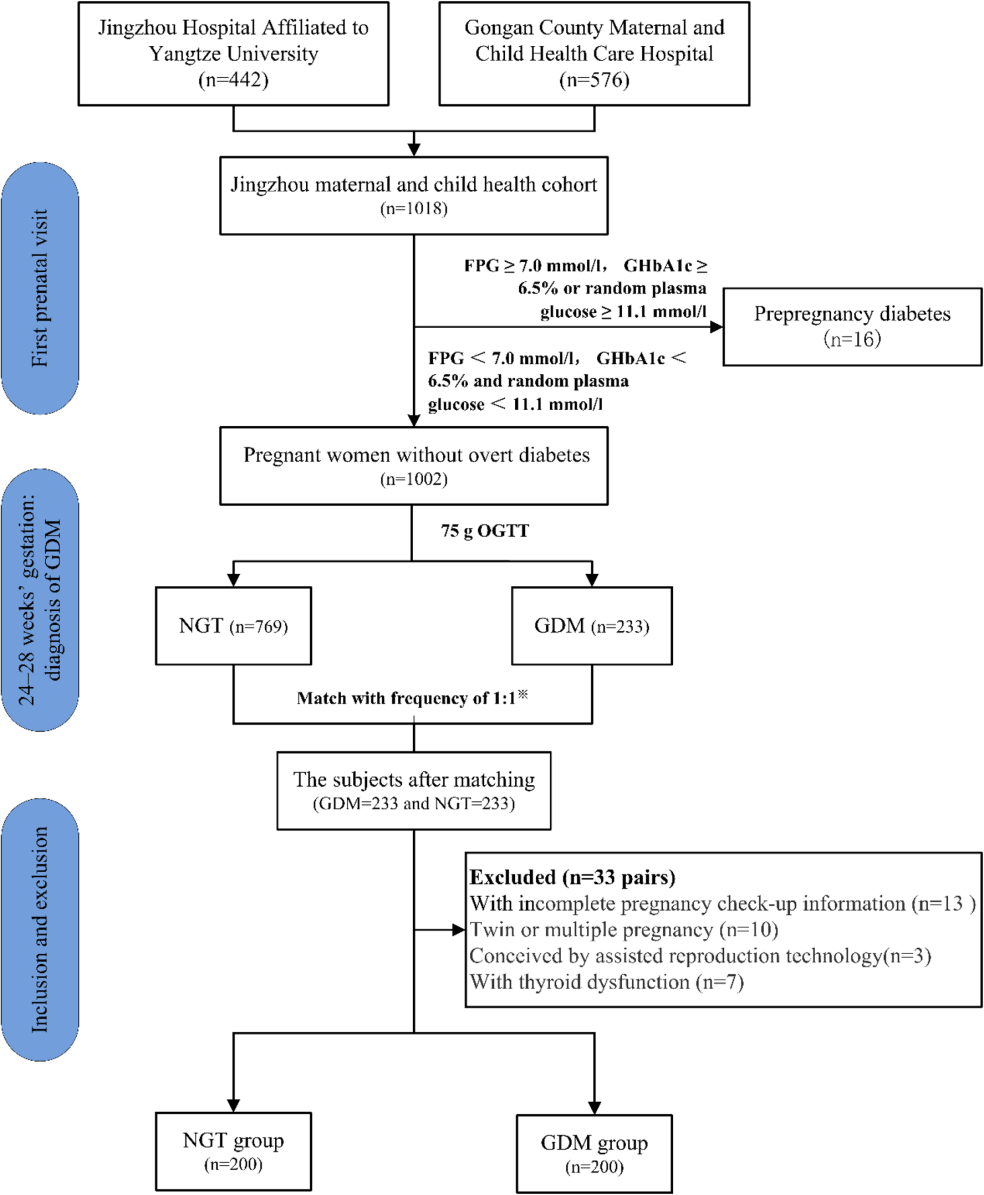
Flow chart for the selection of subjects. *The controls were matched with the cases according to age, basal BMI, hospital and hospital visit date of OGTT

It is a two-center nested case‒control study. All subjects were from the Jingzhou maternal and child health cohort. Pregnant women who registered in Jingzhou Hospital Affiliated to Yangtze University and Gongan County Maternal and Child Health Care Hospital in Hubei Province of China from April 2021 to December 2021, and were willing to undergo routine pregnancy check-ups in these two hospitals and provide informed consent for participation could be recruited into the cohort. All pregnant women were recruited into the cohort on the day of the first pregnancy check-up. The diagnosis of GDM was based upon the Health Industry Standard of the People's Republic of China of “Diagnosis criteria for gestational diabetes mellitus” (WS 331-2011) which adopts recommendations of the diagnostic criteria of the International Association of Diabetes and Pregnancy Study Groups (IADPSG). All pregnant women in the cohort were routinely tested for FPG, glycosylated hemoglobin a1c (GHbA1c), or random plasma glucose at the first prenatal visit to identify prepregnancy diabetes (overt diabetes). Pregnant women were diagnosed as having overt diabetes and excluded from our study when (1) FPG ≥ 7.0 mmol/L (126 mg/dL); (2) GHbA1c ≥ 6.5% (DCCT/UKPDS standardized); or (3) having typical symptoms of hyperglycemia or hyperglycemic crisis and random plasma glucose ≥ 11.1 mmol/L (200 mg/dL) (the values of FPG and GHbA1c need to be rechecked on another day when there are no obvious symptoms of hyperglycemia). All the pregnant women without prepregnancy diabetes underwent a 75 g OGTT at 24–28 gestational weeks. Subjects were diagnosed as GDM when at least one value from the 75 g oral glucose tolerance test (OGTT) was no less than the following levels: FPG 5.1 mmol/L, 1-h plasma glucose (1hPG) 10.0 mmol/L and 2-h plasma glucose (2hPG) 8.5 mmol/L. Pregnant women in the cohort who underwent the 75 g OGTT at 24–28 gestational weeks and met the above diagnostic criteria were included in the case group (GDM group). Age-, basal BMI- and hospital visit date of OGTT-matched normal glucose tolerance (NGT) individuals in the cohort were selected as controls (NGT group) at a frequency of 1:1. When multiple matched controls were available, the subject who matched best was selected. Subjects who met the following criteria were excluded: (1) had other types of diabetes; (2) had heart, liver or kidney disease, hypertension, thyroid dysfunction and polycystic ovary syndrome (PCOS) identified before the diagnosis of GDM (before 24–28 gestational weeks); (3) had twin or multiple pregnancies; (4) conceived by assisted reproduction technology; (5) had taken drugs affecting glucose metabolism during pregnancy; and (6) had incomplete pregnancy check-up information. Finally, a total of 400 pregnant women (200 cases and 200 controls) were included in our study. The flow chart for the selection of subjects is shown in Fig. 1.

Two milliliters of EDTA anticoagulant blood samples and serum samples based upon a 75 g OGTT were collected and then stored at − 80 °C until DNA extraction and insulin measurement. Single nucleotide polymorphism (SNP) genotyping was performed on all 400 subjects, among which insulin levels were measured only in 218 subjects (109 patients and 109 controls).

### Selection of tag SNPs

We did not plan to select tag SNPs with data from public databases, such as the 1000 genome, because we would perform SNP genotyping and identify SNPs that influence GDM susceptibility using direct sequencing. Selecting tag SNPs based on the sequencing and genotyping data from our 400 study subjects is more representative of our study population. Two PCR fragments covering two exons and four introns of the GLP-1R gene, which contain high-density SNPs with a minor allele frequency (MAF) beyond 0.10 in the East Asian population and contain at least one of the few missense variant SNPs reported to be related to the susceptibility or pathogenesis of diabetes were analyzed by Sanger sequencing. Finally, 12 SNPs with MAFs beyond 0.10 in our study population were identified, including 2 missense variant SNPs, 1 synonymous change and 9 intronic variant SNPs. There were 4 SNPs (rs1042044, rs10305478, rs10305479, rs6458093) in fragment 1 (located in 39073424–39074250 Homo sapiens chromosome 6, GRCh38), including the missense variant SNP rs1042044 reported to be associated with susceptibility to T2DM [19], and 8 SNPs (rs910168, rs910167, rs910165, rs910163, rs910162, rs3765468, rs3765467, rs3765466) in fragment 2 (located in 39065201–39066245 Homo sapiens chromosome 6, GRCh38), including the missense variant SNP rs3765467 reported to be associated with insulin secretion, β cell apoptosis and T2DM [15]. The genotypes of the 400 subjects and chromosomal position information of the 12 SNPs were input into Haploview 4.2 software for linkage disequilibrium (LD) analysis and selection of tag SNPs [26]. Finally, Haploview identified one LD block and five tag SNPs (rs10305478 C > T, rs6458093 A > G, rs1042044 A > C, rs3765468 G > A, rs3765467 G > A) that could capture the 12 SNPs. Information for 12 SNPs captured by the 5 tag SNPs is shown in Table 1. Given the strong linkage of the 12 SNPs, 5 tag SNPs rather than 12 SNPs were used in the following analyses.

**Table 1:** Information on the tag SNPs

Tag SNPs	SNPs captured
rs10305478	rs910167, rs910168, rs10305478
rs6458093	rs910165, rs3765466, rs6458093
rs1042044	rs910163, rs1042044, rs910162
rs3765468	rs3765468, rs10305479
rs3765467	rs3765467

### Laboratory measurements

Genomic DNA was isolated following the guidelines of the TIANamp Blood DNA Kit DP348 (Tiangen, China). PCR amplicons were designed to be approximately 1000 bp for optimal Sanger sequencing analysis and included as many SNPs as possible. Two primer pairs were designed for amplification of two selected fragments synthesized by Sangon Biotech (Wuhan) Co., Ltd. The primer sequences of fragment 1 (827 bp) were as follows: forward—5′-CAGGATAGTGAGGATTCAATG-3′, reverse—5′-CACTGTACTTGGCTCTCC-3′. The primer sequences of fragment 2 (1045 bp) were as follows: forward—5′-ACTCAGTGCCAACCTTGTTC-3′, reverse—5′-CCACCGTGTAGATGATGTAGAG-3′. Standard PCRs for the two fragments were performed in a final volume of 25 μL, containing 2 μL (50 ng) of genomic DNA, 2.5 μL 10 × Buffer (Mg^2+^ plus) (Takara), 2 μL dNTPs (Takara), 0.125 μL Taq DNA polymerase (5 U/μL) (Takara), and 1.25 μL forward and reverse primers (20 μM) (Sangon Biotech). The amplification conditions of fragments 1/2 were initial denaturation at 95 °C for 3 min, followed by 35 cycles of 98 °C for 10 s, 64. 4 °C (fragment 1)/63. 3 °C (fragment 2) for 30 s and 72 °C for 1 min. The specificity of the PCR products was verified by electrophoresis in a 2% agarose gel stained with GelRed nucleic acid dye (Biotium) before sequencing. Sanger sequencing was used for SNP genotyping, completed by Sangon Biotech (Wuhan) Co., Ltd.

The levels of glucose were measured by the glucose oxidase method on biochemical analyzers of AU680 and AU5800 (Beckman Coulter, America). The 75 g OGTT-based fasting insulin (FINS), 1-h insulin (1hINS) and 2-h insulin (2hINS) were measured by enzyme-linked immunosorbent assay (ELISA) using Insulin ELISA BS-0783 (Bensheng (Tianjin) Health Technology Co., Ltd., China). The composite (Matsuda) insulin sensitivity index (ISI_Matsuda_) was calculated to assess insulin sensitivity. The 60 min insulinogenic index (IGI_60_) and OGTT-derived disposition index (DI) which have been reported to be more sensitive than the conventional index—homoeostasis model assessment of β cell function (HOMA-β) were calculated to dynamically estimate β cell function [27]. ISI_Matsuda_ was calculated as 10,000/[(FPG × FINS)^1/2^ (GLUmean × INSmean)^1/2^] using fasting, 1-h, and 2-h values of OGTT. IGI_60_ was calculated as (1hINS-FINS)/(1hPG-FPG) and we estimated DI by multiplying IGI_60_ with ISI_Matsuda_ to reflect β cell function adjusting for insulin sensitivity.

### Ethical approval

All subjects provided informed consent for participation, and the study was approved by the ethics committees of Jingzhou Hospital Affiliated to Yangtze University and Gongan County Maternal and Child Health Care Hospital. This study followed the principles of the Declaration of Helsinki.

### Statistical analysis

Normally distributed data are presented as the mean ± SD. Skewed distribution data are displayed as the median (interquartile range). P–P plots were used for normality tests, and the Levene test was used for homogeneity of variance tests. Two-sample t test for continuous variables conforming to normal distribution, Mann‒Whitney U test for continuous variables with nonnormal distribution and Chi-square test or Mann‒Whitney U test for categorical variables were used to evaluate the difference in means and proportions between case and control group. ANOVA and Kruskal‒Wallis tests were used to assess the difference in means of metabolic parameters between different genotypes. Multiple comparisons were conducted by the Bonferroni test. Logistic regression was performed to evaluate the association of GLP-1R gene polymorphisms and GDM risk. *p* < 0.05 was accepted as statistically significant. Analyses were performed with SPSS 25.0 software. Hardy–Weinberg equilibrium (HWE) was evaluated using the SNPStats program (http://bioinfo.iconcologia.net/SNPstats). LD analysis and selection of tag SNPs were calculated using HaploView 4.2 software.

## Results

### Baseline characteristics of participants

To eliminate the effects of confounding factors and keep the baseline characteristics of the GDM and NGT groups as consistent as possible, we matched the two groups according to age, basal BMI, hospital and hospital visiting date of OGTT. Moreover, we collected as much information about the subjects as possible, such as sociodemographic and clinical characteristics, to compare and identify potential confounders. As shown in Table 2, the GDM and NGT groups had comparable age, height, weight, first-trimester BMI, diastolic blood pressure and gestational age at enrollment. There was also no significant difference between the two groups in the component ratios of occupation, education level, history of pregnancy and parturition, domicile address and nature. However, systolic blood pressure in the GDM group was significantly higher than that in the NGT group (*p* = 0.006), which would be considered a confounder to adjust for covariates in subsequent analyses. Moreover, although age and first-trimester BMI showed no difference between the two groups, they were also used as confounders to adjust for covariates to eliminate their weak influence.

**Table 2:** Baseline characteristics of the study population

Variables	GDM (n = 200)	NGT (n = 200)	*p*
Age (year)	29.89 ± 4.07	29.67 ± 3.83	0.578
Height (cm)	160.25 ± 4.61	160.47 ± 5.09	0.651
Weight (kg)	58.49 ± 9.59	58.43 ± 9.63	0.951
First-trimester BMI (kg/m^2^)	22.76 ± 3.50	22.66 ± 3.44	0.783
Systolic blood pressure (mmHg)	112.50 ± 10.63	109.69 ± 9.81	**0.006**
Diastolic blood pressure (mmHg)	71.37 ± 9.07	71.11 ± 8.06	0.766
Gestational age at enrollment (week)	11.64 ± 3.27	12.12 ± 4.32	0.209
Occupation			0.233
Staff of administrative, enterprise or institution	118 (59.0%)	128 (64.0%)	
Freelancer	37 (18.5%)	38 (19.0%)	
Other practitioner	10 (5.0%)	4 (2.0%)	
Not stated	35 (17.5%)	30 (15.0%)	
Education			0.172
Primary school	1 (0.5%)	1 (0.5%)	
Junior middle school	18 (9.0%)	14 (7.0%)	
High school or technical school	119 (59.5%)	122 (61.0%)	
College or above	26 (13.0%)	36 (18.0%)	
Not stated	36 (18.0%)	27 (13.5%)	
Permanent Address			1.000
Local resident	197 (98.5%)	197 (98.5%)	
Nonlocal resident	3 (1.5%)	3 (1.5%)	
The nature of household registration			0.188
Agriculture	192 (96.0%)	186 (93.0%)	
Nonagriculture	8 (4.0%)	14 (7.0%)	
Gravidity			0.648
1	78 (39.0%)	73 (36.5%)	
2–4	108 (54.0%)	113 (56.5%)	
≥ 5	14 (7.0%)	14 (7.0%)	
Parity			0.153
0	114 (57.0%)	99 (49.5%)	
1	79 (39.5%)	94 (47.0%)	
≥ 2	7 (3.5%)	7 (3.5%)	

The *p* values were calculated using Two-sample t test or chi-square test according to the type of variables

Variables with statistical significance are shown in boldface

*GDM* gestational diabetes mellitus, *NGT* normal glucose tolerance, *BMI* body mass index

### Relationship between GLP-1R polymorphisms and GDM risk

The genotype distributions of the 5 tag SNPs showed no deviation from HWE in cases and controls. Table 3 shows the genotype frequency distribution of the 5 tag SNPs in the GDM and NGT groups. We analyzed five genetic models for each SNP. Logistic regression in Table 3 showed that rs6458093 was associated with an approximately 1.54-fold increased risk of GDM under the dominant model (*p* = 0.044), and this effect remained nominally significant after adjusting for age, first-trimester BMI and systolic blood pressure (*p* = 0.049).

**Table 3:** Association between GLP-1R gene polymorphisms and gestational diabetes mellitus susceptibility

SNPs	Genotypes	NGT (n = 200)	GDM (n = 200)	Codominant		Dominant		Recessive		Overdominant		Log-additive		Allele	
				OR (95% CI)	*p*	OR (95% CI)	*p*	OR (95% CI)	*p*	OR (95% CI)	*p*	OR (95% CI)	*p*	OR (95% CI)	*p*
rs3765468	GG	133	121	1.00		1.30 (0.86–1.95)	0.213	1.67 (0.68–4.12)	0.267	1.18 (0.77–1.80)	0.450	1.28 (0.91–1.79)	0.150	1.29 (0.92–1.82)	0.140
G > A	GA	59	66	1.23 (0.80–1.89)	0.345										
	AA	8	13	1.79 (0.72–4.46)	0.214										
rs3765467	GG	124	120	1.00		1.09 (0.73–1.63)	0.682	1.60 (0.61–4.23)	0.339	1.00 (0.66–1.51)	1.00	1.13 (0.80–1.58)	0.490	1.12 (0.80–1.57)	0.493
G > A	GA	69	69	1.03 (0.68–1.57)	0.878										
	AA	7	11	1.62 (0.61–4.33)	0.332										
rs1042044	AA	49	39	1.00		1.34 (0.83–2.16)	0.228	1.07 (0.70–1.65)	0.743	1.15 (0.78–1.70)	0.484	0.88 (0.67–1.16)	0.360	1.14 (0.86–1.51)	0.357
A > C	AC	93	100	1.35 (0.81–2.24)	0.244										
	CC	58	61	1.32 (0.76–2.30)	0.324										
rs10305478	CC	157	165	1.00		0.77 (0.47–1.27)	0.313	0.25 (0.03–2.22)	0.212	0.85 (0.51–1.41)	0.518	0.75 (0.47–1.18)	0.210	0.74 (0.47–1.17)	0.203
C > T	CT	39	34	0.83 (0.50–1.38)	0.472										
	TT	4	1	0.24 (0.03–2.15)	0.201										
rs6458093	AA	76	57	1.00		**1.54 (1.01**–**2.34)**	**0.044**	1.21 (0.74–1.97)	0.454	1.30 (0.88–1.93)	0.193	1.28 (0.97–1.68)	0.080	1.29 (0.98–1.71)	0.075
A > G	AG	87	100	1.53 (0.98–2.40)	0.062	**1.53 (1.00**–**2.34)**^**a**^	**0.049**^**a**^								
	GG	37	43	1.55 (0.89–2.71)	0.124										

*GDM* gestational diabetes mellitus, *NGT* normal glucose tolerance, *GLP-1R* glucagon-like peptide-1 receptor

^a^Adjusted for age, first-trimester BMI and systolic blood pressure. The unmarked *p* and OR values were crude. The p and OR values were calculated using unconditional binary logistic regression. Variables with statistical significance are shown in boldface. Codominant: mutant homozygous genotype vs heterozygous genotype vs wild homozygous genotype; Dominant: heterozygous genotype + mutant homozygous genotype vs wild homozygous genotype; Recessive: mutant homozygous genotype vs wild homozygous genotype + heterozygous genotype; Overdominant: heterozygous genotype vs wild homozygous genotype + mutant homozygous genotype; Log-additive: additive effects of risk allele

Age and BMI are recognized as the most important environmental risk factors for GDM, so we performed analyses stratified by age and BMI. As shown in Table 4, genotype GA of rs3765467 decreased GDM risk among subjects older than 35 years old after adjusting for age, first-trimester BMI and systolic blood pressure (*p* = 0.037). Genotype AG of rs6458093 was associated with increased GDM risk only among pregnant women younger than 35 years old after adjusting for age, first-trimester BMI and systolic blood pressure (*p* = 0.029). Moreover, rs6458093 was associated with increased GDM risk under the dominant model (AG + GG vs. AA) among pregnant women younger than 35 years old (*p* = 0.024) and with BMI no less than 24 (*p* = 0.041) after adjusting for confounders.

**Table 4:** Association between GLP-1R polymorphisms and risk of GDM after stratification by age and BMI

SNP	Genotype	Age < 35 years (n = 354)				Age ≥ 35 years (n = 46)				BMI < 24 (n = 280)				BMI ≥ 24 (n = 120)			
		NGT (n = 182)	GDM (n = 172)	OR (95%CI)	*p*	NGT (n = 18)	GDM (n = 28)	OR (95%CI)	*p*	NGT (n = 142)	GDM (n = 138)	OR (95%CI)	*p*	NGT (n = 58)	GDM (n = 62)	OR (95%CI)	*p*
rs3765468	GG	121	105	1.00		12	16	1.00		92	78	1.00		41	43	1.00	
	GA	54	56	1.19 (0.76, 1.89)	0.444	5	10	1.50 (0.41, 5.55)	0.544	44	49	1.31 (0.79, 2.18)	0.291	15	17	1.08 (0.48, 2.44)	0.852
	AA	7	11	1.81 (0.68, 4.84)	0.236	1	2	1.50 (0.12, 18.54)	0.751	6	11	2.16 (0.77, 6.12)	0.146	2	2	0.95 (0.13, 7.09)	0.963
	GA + AA	61	67	1.27 (0.82, 1.95)	0.288	6	12	1.50 (0.44, 5.15)	0.519	50	60	1.42 (0.87, 2.29)	0.157	17	19	1.07 (0.49, 2.33)	0.873
rs3765467	GG	116	100	1.00		8	20	1.00		87	83	1.00		37	37	1.00	
	GA	60	65	1.26 (0.81, 1.95)	0.310	9	4	**0.18 (0.04, 0.75)**	**0.018**	50	52	1.09 (0.67, 1.78)	0.731	19	17	0.90 (0.40, 1.99)	0.79
								**0.20 (0.04, 0.91)**^**a**^	**0.037**^**a**^								
	AA	6	7	1.35 (0.44, 4.16)	0.597	1	4	1.60 (0.15, 16.61)	0.694	5	3	0.63 (0.15, 2.72)	0.534	2	8	4.00 (0.80, 20.11)	0.093
	GA + AA	66	72	1.27 (0.83, 1.94)	0.281	10	8	0.32 (0.09, 1.11)	0.072	55	55	1.05 (0.65, 1.69)	0.848	21	25	1.19 (0.57, 2.49)	0.643
rs1042044	AA	46	32	1.00		3	7	1.00		32	28	1.00		17	11	1.00	
	AC	86	90	1.50 (0.88, 2.58)	0.138	7	10	0.61 (0.12, 3.23)	0.563	69	68	1.13 (0.61, 2.07)	0.701	24	32	2.06 (0.82, 5.20)	0.125
	CC	50	50	1.44 (0.79, 2.61)	0.234	8	11	0.59 (0.12, 3.01)	0.525	41	42	1.17 (0.60, 2.28)	0.642	17	19	1.73 (0.63, 4.70)	0.285
	AC + CC	136	140	1.48 (0.89, 2.46)	0.131	15	21	0.60 (0.13, 2.71)	0.506	110	110	1.14 (0.65, 2.03)	0.647	41	51	1.92 (0.81, 4.56)	0.138
rs10305478	CC	144	143	1.00		13	22	1.00		113	114	1.00		44	51	1.00	
	CT	34	28	0.83 (0.48, 1.44)	0.506	5	6	0.71 (0.18, 2.80)	0.623	26	23	0.88 (0.47, 1.63)	0.677	13	11	0.73 (0.30, 1.79)	0.492
	TT	4	1	0.25 (0.03, 2.28)	0.220	0	0	–	–	3	1	0.33 (0.03, 3.22)	0.341	1	0	0.00	1.00
	CT + TT	38	29	0.77 (0.45, 1.31)	0.335	5	6	0.71 (0.18, 2.80)	0.623	29	24	0.82 (0.45, 1.50)	0.52	14	11	0.68 (0.28, 1.65)	0.39
rs6458093	AA	72	48	1.00		4	9	1.00		51	41	1.00		25	16	1.00	
	AG	77	88	**1.71 (1.07, 2.77)**	**0.027**	10	12	0.533 (0.13, 2.27)	0.394	64	67	1.30 (0.76, 2.22)	0.333	23	33	2.24 (0.99, 5.11)	0.055
				**1.71 (1.06, 2.75)**^**a**^	**0.029**^**a**^												
	GG	33	36	1.64 (0.90, 2.98)	0.106	4	7	0.78 (0.14, 4.27)	0.772	27	30	1.38 (0.71, 2.68)	0.339	10	13	2.03 (0.72, 5.72)	0.180
	AG + GG	110	124	**1.69 (1.08, 2.64)**	**0.021**	14	19	0.60 (0.15, 2.36)	0.603	91	97	1.33 (0.80, 2.19)	0.270	33	46	**2.18 (1.01, 4.71)**	**0.048**
				**1.68 (1.07, 2.63)**^**a**^	**0.024**^**a**^											**2.32 (1.04, 5.18)**^**a**^	**0.041**^**a**^

*GDM* gestational diabetes mellitus, *NGT* normal glucose tolerance, *BMI* body mass index, *GLP-1R* glucagon-like peptide-1 receptor

^a^Adjusted for age, first-trimester BMI and systolic blood pressure. The unmarked p and OR values were crude. The p and OR values were calculated using unconditional binary logistic regression. Variables with statistical significance are shown in boldface

### Effects of GLP-1R polymorphisms on glucose metabolism and beta cell function

**Fig. 2:**
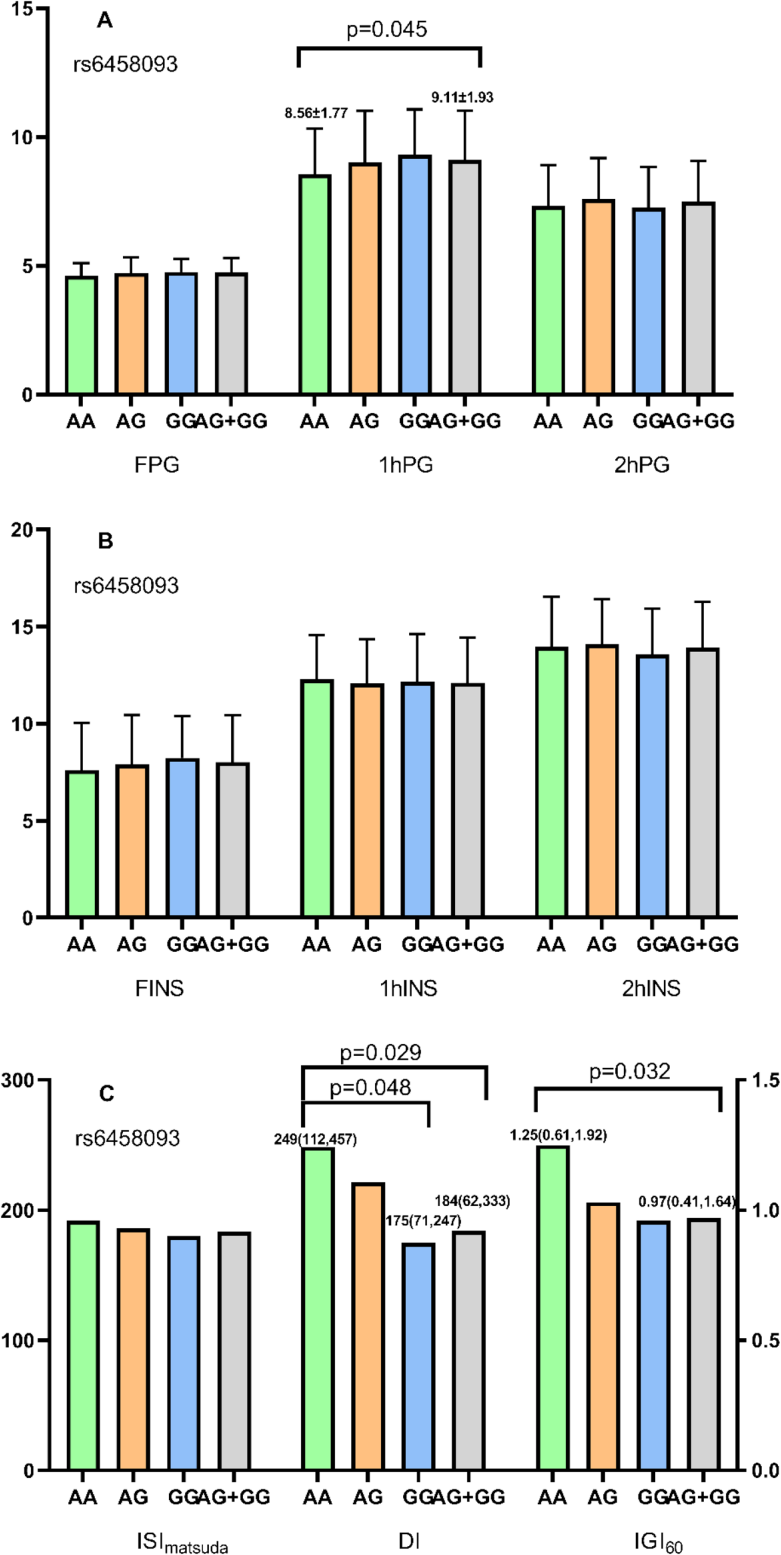
Effects of rs6458093 on glucose metabolism. **A** Effects of rs6458093 on glucose parameters. **B** Effects of rs6458093 on insulin parameters. **C** Effects of rs6458093 on insulin sensitivity and beta cell function. FPG: fasting plasma glucose; 1hPG: 1-h plasma glucose; 2hPG: 2-h plasma glucose; FINS: fasting insulin; 1hINS: 1-h insulin; 2hINS: 2-h insulin; ISI_matsuda_: Matsuda insulin sensitivity index; DI: disposition index; IGI_60_: 60 min insulinogenic index

**Fig. 3:**
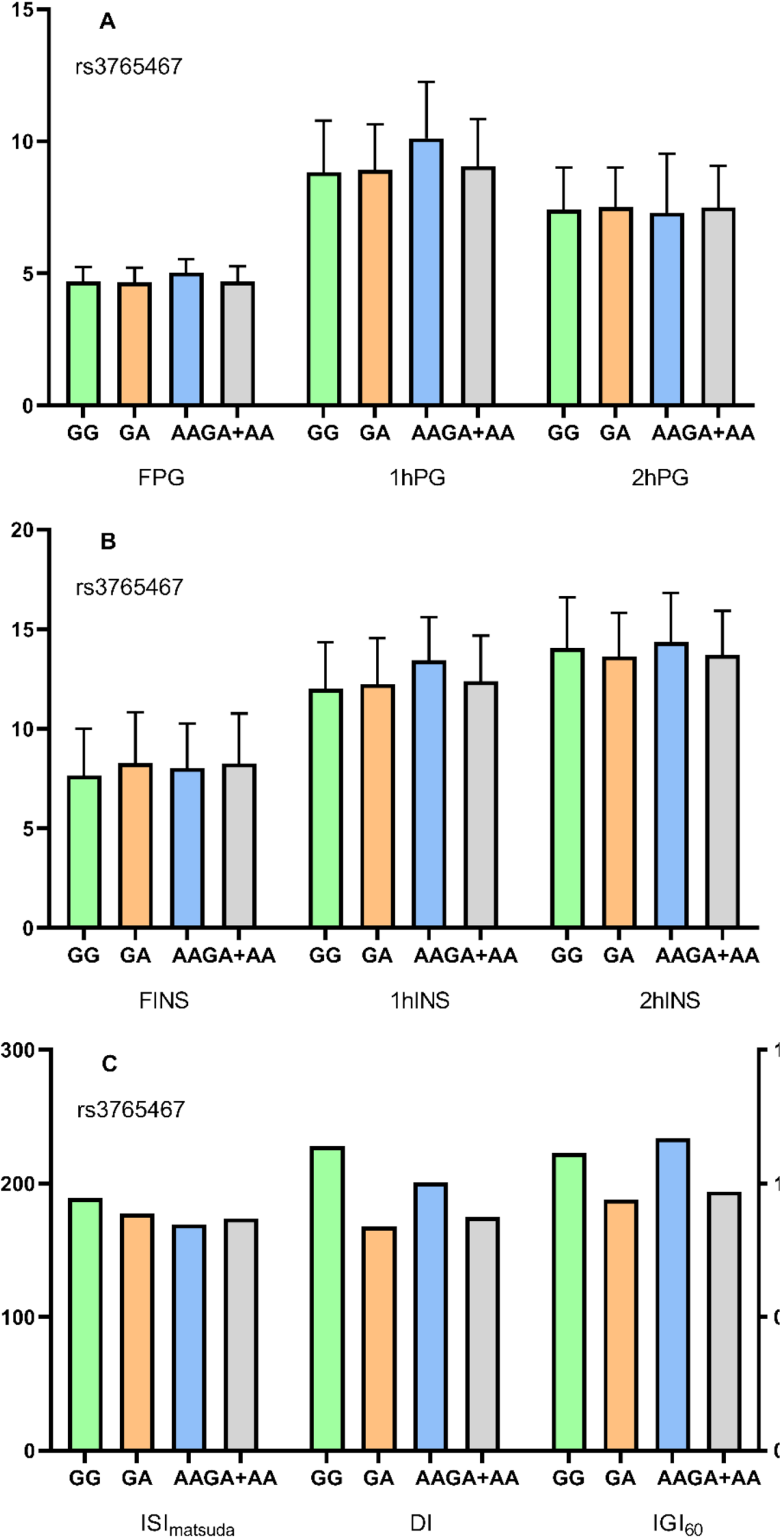
Effects of rs3765467 on glucose metabolism. **A** Effects of rs3765467 on glucose parameters. **B** Effects of rs3765467 on insulin parameters. **C** Effects of rs3765467 on insulin sensitivity and beta cell function. FPG: fasting plasma glucose; 1hPG: 1-h plasma glucose; 2hPG: 2-h plasma glucose; FINS: fasting insulin; 1hINS: 1-h insulin; 2hINS: 2-h insulin; ISI_matsuda_: Matsuda insulin sensitivity index; DI: disposition index; IGI_60_: 60 min insulinogenic index

We compared and estimated metabolic parameters of different genotypes, including FPG, 1hPG, 2hPG, FINS, 1hINS, 2hINS, ISI_matsuda_, IGI_60_ and DI. As shown in Fig. 2, genotype AG + GG of rs6458093 (A > G) showed significantly lower IGI_60_ and DI than genotype AA, as well as significantly higher 1hPG. No significant difference in metabolic parameters was found among different genotypes of rs3765467 (Fig. 3).

## Discussion

The prevalence of GDM continues to increase steadily [28]. However, the etiology and pathogenesis of GDM remain vague due to its complexity under the combined action of environmental and genetic factors. Currently, environmental risk factors for GDM, such as age, BMI [29], sleep disorders [30], and polycystic ovary syndrome [31], have been well explained, but genetic risk factors still have not been well defined.

In this study, we conducted a two-center nested case‒control study based on the important role of GLP-1R in glucose homeostasis [32, 33] aimed to explore genetic risk factors for GDM. GLP-1R is a kind of G protein-coupled receptors [34]. When GLP-1 binds to GLP-1R, adenylate cyclase (AC) is activated by G protein to increase the intracellular concentration of cAMP. On the one hand, increased cAMP leads to the closure of K^+^ channels on the cytomembrane, depolarization of cell, opening of voltage-dependent Ca^2+^ channels, influx of extracellular Ca^2+^, increase of intracellular Ca^2+^ concentration, and synthesis and release of insulin finally. On the other hand, the increased cAMP activates protein kinase A (PKA) to phosphorylate related proteins, which further stimulates the transcription and translation of insulin gene finally [32, 34–36]. It was reported that very little incretin effect remained and glucose tolerance was pathologically impaired in double incretin receptors knockout mice [37, 38], illustrating that GLP-1R dysfunction affected the incretin effect producing insulin. Besides stimulating insulin synthesis and secretion, GLP-1R also mediates GLP-1 to stimulate β cell proliferation and neogenesis and inhibit β cell apoptosis [39]. Furthermore, abundant evidence suggests that SNPs of GLP-1R, such as rs10305420, rs3765467 and rs6923761, lead to GLP-1R dysfunction, subsequently affecting the binding of GLP-1 or GLP-1R agonists (GLP-1RAs) to GLP-1R, and ultimately affecting the hypoglycemic effects of GLP-1RAs and inhibitor of dipepdityl peptidase-4 (DPP-4) (the enzyme responsible for incretin degradation) [40–42]. In the same way, SNPs of GLP-1R impaired receptor function, subsequently affecting β cell insulin secretion function and ultimately affecting the efficacy of Roux-en-Y gastric bypass [15]. The above studies indicate that GLP-1R gene polymorphism is related to β cell insulin secretion function, which may affect glucose metabolism and susceptibility of GDM.

In our study, we identified two GLP-1R tag SNPs, rs6458093 and rs3765467, associated with GDM susceptibility, and rs6458093 also affected β cell function and postprandial glucose metabolism. The tag SNP rs6458093 was located in the intron region that may influence gene function by changing the level, location, stability, or timing of gene expression [43], directly influencing the quality or quantity of the gene product GLP-1R. Tag SNP rs3765467 is a missense mutation resulting in substitution of glutamine for arginine at position 131, directly influencing the function of GLP-1R [14]. As mentioned above, rs6458093 and rs3765467 might affect the interaction of GLP-1 and GLP-1R by affecting the quality or quantity of GLP-1R, then affecting β cell insulin secretion function, and ultimately altering glucose metabolism and susceptibility of GDM.

As rare missense variant SNPs with a high frequency of MAF in GLP-1R, rs1042044 and rs3765467 are often the focus of research. Although rs1042044 had nothing to do with insulin secretion [15], it was reported to be associated with susceptibility of T2DM in a Chinese literature [19] and the risk of papillary thyroid cancer among the Egyptian population in a recent study [44]. Moreover, rs1042044 was also differentially associated with brain functional connectivity in individuals with low versus high severity of alcohol use [45]. Yapici-Eser et al. [46] suggested a possible association of rs1042044 with anhedonia but no association with depression diagnosis. However, a correlation between rs1042044 and GDM was not found in our study. For rs3765467, Korean patients with T2DM carrying mutant allele A (GA/AA) showed a significantly better hypoglycemic effect to DPP-4 inhibitors than those with genotype GG [47], indicating that mutant genotype GA/AA could improve the function of GLP-1R mediating insulin secretion, while the major genotype GG might be a risk factor for disease. Research from the Mayo Clinic also suggested that subjects with genotype GA of rs3765467 exhibited a 100% increase in the total insulin secretion response to infused GLP-1 in the presence of hyperglycemia compared with subjects carrying the GG genotype [14]. Nishiya et al. [48] also found that the GG genotype was a significant risk factor for decreased insulin secretion. Moreover, rs3765467 was reported to be associated with T2DM risk [16]. Although no evidence in our study showed that rs3765467 affected β cell function and glucose metabolism, it was found that the GA genotype of rs3765467 was related to a decreased risk of GDM among pregnant women older than 35 years. This inconsistent result might be explained by the fact that rs3765467 was mainly related to a decreased risk of GDM among pregnant women older than 35 years. Regrettably, in our study, it was not feasible to compare metabolic parameters of different genotypes stratified by age due to the small sample size of subjects older than 35 years.

To our knowledge, rs6458093, rs10305478 and rs3765468 have not been reported to be related to disease or insulin secretion, so we know little about their role. However, our study found that rs6458093 was not only associated with GDM susceptibility but also affected postprandial glucose homeostasis and β cell function. The findings again implied that genetic variations in GLP-1R may affect susceptibility to GDM by affecting β cell function, especially postprandial insulin secretion and glucose homeostasis, which could be explained by the fact that incretin hormones mainly mediate postprandial insulin secretion and glucose homeostasis [49].

With a high MAF in the European population, the missense variant SNP rs6923761 is the most popular study locus. It was reported to be associated with a glucose response to a mixed meal [50], a smaller glycemic response to treatment with DPP-4 inhibitors [42, 51], delayed gastric-emptying in obese patients treated with liraglutide (GLP-1RAs) or exenatide (GLP-1 analog) [52], weight loss and metabolic change in diabetic patients treated with liraglutide [53] and so on. Despite many studies supporting the hypothesis that rs6923761 may be associated with GDM susceptibility and glucose metabolism, rs6923761 was not included in our study due to the low MAF of < 0.01 in the East Asian population. Regrettably, the missense variant SNP rs10305420 was not included in our study because it was localized in a fragment with a low SNP density. Future studies exploring the relationship between rs10305420 and GDM susceptibility are reasonable, as rs10305420 was reported to be associated with reduced glycemic response and less weight loss response to GLP-1R agonists [54].

Furthermore, our findings also have some enlightenment for research on T2DM. Kwak et al. even suggested the identification of genetic risk loci in GDM as a complementary approach to improve our insight into the genetics of T2DM given the similar genetic basis between GDM and T2DM [55]. It remains to be determined whether rs6458093 and rs3765467 also play roles in the development of T2DM. Moreover, GLP-1-based therapeutics have been rapidly developed and used clinically for the treatment of T2DM. Despite many advantages, individual differences in treatment outcomes are obvious. Some SNPs in GLP-1R, such as rs6923761 [42, 50], rs10305420 [41], and rs3765467 [40], have been reported to lead to individual differences in these therapeutics. The strong association between GLP-1R gene polymorphisms and the hypoglycemic effects of DPP-4 inhibitors and GLP-1RAs makes the GLP-1R gene a candidate gene for precision medicine in diabetes [56]. Future studies are needed to determine whether rs6458093 accounts for interindividual differences in response to GLP-1-based therapy.

Our study also has some limitations. First, although there is a large population in our country and there are 1018 pregnant women in our cohort, the sample size in our study was limited to some extent due to our study design (strict inclusion and exclusion criteria, and strict 1:1 frequency matching between the case group and control group based on a nested case‒control study aimed to minimize the influence of confounding factors as much as possible), so subgroup analyses and stratification analyses of metabolic parameters for rs3765467 could not be performed. Second, lifestyle was not considered in our study since GDM is a lifestyle-related disease. Third, none of the reported *p* values in this study were corrected for multiple testing. The *p* values of < 0.01 were significant after Bonferroni correction for 5 SNPs. Fourth, we can only use some indices calculated to estimate β cell function due to the particular period of pregnancy, while the gold standard for assessing β cell function remains the glucose clamp study. Each calculated index has some limitations.

In conclusion, our study identified two tag SNPs of GLP-1R nominally associated with GDM susceptibility, and one of them affected beta cell function and postprandial glucose metabolism, providing evidence for the etiology and genetic study of GDM and some inspirations for T2DM.

## Data Availability

The datasets used in this study are available from the corresponding author on reasonable request.

## Abbreviations

GDM: Gestational diabetes mellitus
T2DM: Type 2 diabetes mellitus
β cell: Beta cell
NGT: Normal glucose tolerance
GLP-1: Glucagon-like peptide-1
GLP-1R: Glucagon-like peptide-1 receptor
SNP: Single nucleotide polymorphism
MAF: Minor allele frequency
LD: Linkage disequilibrium
OGTT: Oral glucose tolerance test
FPG: Fasting plasma glucose
1hPG: 75 g OGTT-based 1-h plasma glucose
2hPG: 75 g OGTT-based 2-h plasma glucose
FINS: Fasting insulin
1hINS: 75 g OGTT-based 1-h insulin
2hINS: 75 g OGTT-based 2-h insulin
ELISA: Enzyme-linked immunosorbent assay
ISI_Matsuda_: Matsuda insulin sensitivity index
IGI_60_: Insulinogenic index at 60 min
DI: 75 g OGTT-derived disposition index
HWE: Hardy–Weinberg equilibrium
BMI: Body mass index
GLP-1RAs: Glucagon-like peptide-1 receptor agonists
DPP-4: Dipepdityl peptidase-4
GHbA1c: Glycosylated hemoglobin a1c
